# Molecular characterization of bovine tuberculosis strains in two slaughterhouses in Morocco

**DOI:** 10.1186/s12917-017-1165-6

**Published:** 2017-08-25

**Authors:** Hind Yahyaoui-Azami, Hamid Aboukhassib, Mohammed Bouslikhane, Jaouad Berrada, Soukaina Rami, Miriam Reinhard, Sebastien Gagneux, Julia Feldmann, Sonia Borrell, Jakob Zinsstag

**Affiliations:** 1 Department of Epidemiology and Public Health, Swiss Tropical and Public Health Institute, Socinstrasse 57, 4002 Basel, Switzerland; 2 University of Basel, Basel, Switzerland; 3 Department of Pathology and Veterinary Public Health, Hassan II Agronomy and Veterinary Institute, Rabat, Morocco; 4 Département de Biologie, Equipe Physiopathologies Nutritionnelles et Toxicologie, Faculté des Sciences UCD, El Jadida, Morocco; 5 Office Nationale de Sécurité Sanitaire des produits Alimentaires, Rabat, Morocco

**Keywords:** *Mycobacterium bovis*, Bovine tuberculosis, Morocco, Cattle, Slaughterhouse, Spoligotype, PCR

## Abstract

**Background:**

Bovine tuberculosis (BTB) is caused by *Mycobacterium bovis*, which belongs to the *Mycobacterium tuberculosis* complex. *Mycobacterium bovis* have been described to be responsible of most cases of bovine tuberculosis. Although *M. tuberculosis*, *M. africanum* and non-complex mycobacteria were isolated from cattle.

In Morocco, so far, no molecular studies were conducted to characterize the strains responsible of BTB. The present study aims to characterize *M. bovis* in Morocco.

The present study was conducted in slaughterhouses in Rabat and El Jadida. Samples were collected from 327 slaughtered animals with visible lesions suggesting BTB.

**Results:**

A total of 225 isolates yielded cultures, 95% (*n* = 215) of them were acid-fast (AF). Sixty eight per cent of the AF positive samples were confirmed as tuberculous mycobacteria (*n* = 147), 99% of these (*n* = 146) having RD9 and among the latter, 98% (*n* = 143) positive while 2% (*n* = 3) negative for RD4

A total of 134 samples were analyzed by spoligotyping of which 14 were in cluster and with 41 different spoligotypes, ten of them were new patterns (23%). The most prevalent spoligotypes were SB0121, SB0265, and SB0120, and were already identified in many other countries, such as Algeria, Spain, Tunisia, the United States and Argentina.

**Conclusion:**

The shared borders between Algeria and Morocco, in addition to the previous importation of cattle from Europe and the US could explain the similarities found in *M. bovis* spoligotypes. On the other hand, the desert of Morocco could be considered as an efficient barrier preventing the introduction of BTB to Morocco from West Central and East Africa. Our findings suggest a low level endemic transmission of BTB similar to other African countries. However, more research is needed for further knowledge about the transmission patterns of BTB in Morocco.

## Background

Bovine tuberculosis (BTB) is a chronic granulomatous caseous-necrotising inflammatory disease that mainly affects the lungs and their draining lymph nodes, it can also affect other organs, depending on the route of infection [1–3]. BTB is caused by *Mycobacterium bovis*, which belongs to the *Mycobacterium tuberculosis* complex (MTBC). *M. bovis* has the particular ability to infect a wide range of host species other than cattle (livestock, wildlife and pets) and humans, [3, 4], however, cattle remain the most important reservoir for *M. bovis* [5]. There exist important wildlife reservoirs like the badger (*Meles meles*), the Possum (*Trichosurus vulpecula*) [4].

*M. bovis* can be transmitted between animals by inhalation of aerosols and the ingestion of contaminated food. The transmission is enhanced by many risk factors, mostly related to intensive livestock system [6]. The transmission of *M. bovis* to humans occurs by consumption of unpasteurized infected raw milk and by the contact with infected cattle [7].

While BTB is still occurring in some developed countries in low prevalence [8–10] because of a wildlife reservoir [11] i.e. badgers in the UK [12], this zoonosis is endemic in many of the developing countries, who lack the financial resources to control this disease. Bovine tuberculosis is highly prevalent in many African countries [13, 14], where it causes economic losses by its effects on animal health and productivity and by international trade restrictions. [15]. Bovine tuberculosis is also a zoonosis and, consequently, considered as a public health issue [1, 16, 17].

In Morocco, the agriculture sector is of key importance to the economy, representing approximately 14% of total gross domestic product (GDP) (75bn MAD/ €6.6bn) and approximately 7% of exports (2009). Livestock represents 38% of the total agriculture sector GDP [18]. Both extensive and intensive livestock production systems exist in Morocco, with local, crossbred and imported breeds, mostly Holstein. Local breeds have been shown to be more resistant to the disease [14, 19]. Bovine tuberculosis is an endemic zoonosis in Morocco. The last national survey based on skin tuberculin test was conducted in 2004 and showed an individual prevalence of 18% and a herd prevalence of 33% [20]. Furthermore, a cross-sectional tuberculin study was conducted in the Sidi Kacem area in Morocco in 2012 showing an individual prevalence of 20.4% and a herd prevalence of 57.7% [19].

Officially, skin test and slaughter is the current control strategy applied in Morocco; however this strategy is not fully applied as it is not respected; in addition, there is no systematic BTB screening of cattle at a national level [21].

In a review published by Muller et al. in 2013, the proportion of zoonotic human tuberculosis (TB) among all TB cases was estimated at 1.4% for the non-African countries, and at 2.8% in Africa [22]. The highest prevalence were found to be 13.8% and 7% respectively in Mexico [23] and in Uganda [24]. However, the World Health Organisation states a worldwide median prevalence of 3.1% of *M. bovis* among human TB patients [25]. Even if those prevalence values are mostly low, Muller et al., Pérez-Lago et al. and Navarro, and García-de-Viedma highlight the major consequences of TB due to *M. bovis* on certain groups of the population, and report a potential underestimation of the prevalence of zoonotic human TB [22, 25].

The phylogeny of MTBC showed recently that the strains found in animals belong to a single lineage which showed the deletion of the “Region of Difference” 9 (RD9) [22, 26]. Indeed, *M. bovis* is the most recent strain in his lineage showing the deletion of RD4 [27].

Molecular deletion typing had been found to be an important tool to differentiate *M. bovis* from the other strains of the MTBC [28]. Pattern of presence or absence of these RDs would allow a discrimination among MTBC strains [28–30].

Currently there is no data available in Morocco about the molecular characterization of BTB and the prevalence of MTBC among slaughtered cattle. The aim of the present study was to characterize the strains of MTBC which are responsible for BTB among the slaughtered cattle in Morocco.

## Methods

### Study area and sample collection

The study was conducted in two slaughterhouses in Morocco, one in Rabat and another in EL Jadida, which are two coastal cities separated by 200 km. The cattle slaughtered in these two slaughterhouses come from the rural areas surrounding the two cities and also from many other areas of the country. Individual information of every animal, such as gender, age, breed, and the possible origin were recorded in our database, as well as the date of sampling. The sample collection was performed in Rabat from March to July 2015 and in El Jadida from June 2014 to April 2015.

The samples were conveyed to the Veterinary and Agriculture Institute (IAV) in Rabat and stored in −20 °C until their treatment.

### Tissue preparation, culture

Prior to treatment, samples were thawed overnight at 4 °C. Subsequently, samples were desiccated to remove adipose tissue, and 5 g of the desiccated lesions were mixed with sterilized sand and 10 ml of phenol red. The solution (7.5 ml) was placed in a 15 ml conic tube, 2.5 ml of NaOH 1 N was added at room temperature for 10 min, and then HCl 6 N was added for sample neutralization. As a final step, the tube was centrifuged for 25 min at 3500 rpm.

The supernatant was discarded and pellet was distributed in two of the four pre-tested culture media: Lowenstein-Jensen (LJ), LJ with glycerol (LJG) or pyruvate (LJP) and Herrold according to the availability in the laboratory. Cultures were incubated for 12 weeks at 37 °C and observed daily for growing colonies during the first week then weekly from the second week onwards.

All the grown cultures were deactivated by adding a loopful of mycobacterium colonies to 1 ml of sterilized water contained in small tubes. The samples were then inactivated for 1 h at 90 °C.

### Determination of MTBC and deletion typing

*Mycobacterium* molecular characterization was performed using Multiplex polymerase chain reaction (PCR). The PCR was performed in the TB laboratory at Swiss TPH. We performed first MrpoB PCR in order to differentiate MTC and NTM as described earlier [31]. Deletion analysis by PCR was used to differentiate *M. bovis* and *M. tuberculosis* from other species of the MTBC by assessing the presence or absence of Regions of Difference 9 and 4 (RD). The analysis was carried out as previously described [13, 28].

### Spoligotyping

Spoligotyping was performed as previously described [32]. Spoligotyping patterns were defined according to the SITVIT WEB database [33] and to *Mycobacterium bovis* molecular typing database [34]. All the patterns which were not found in the two databases were submitted as new patterns; new spoligotype numbers were assigned to them.

## Results

### Cattle information

In the present study, a total number of 8658 animals were examined. Three hundred and twenty seven animal presented gross visible lesions (3.7%) and were cultured, 66% (*n* = 215) of the total sampled animals were analysed by Ziehl-Neelsen(ZN), 68% (*n* = 147) of the latter were ZN positive and heat-killed for further molecular typing.

**Fig. 1:**
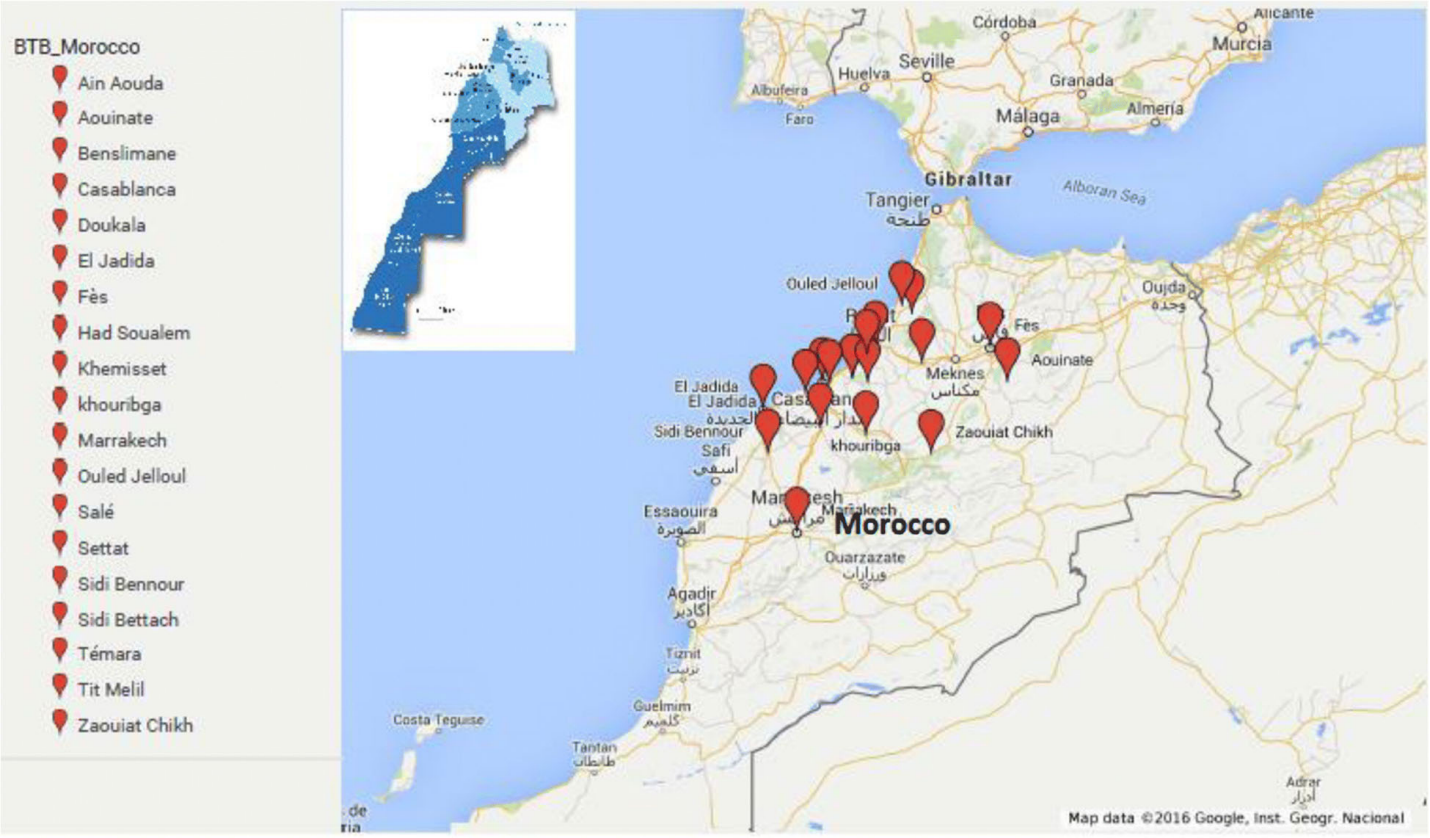
Geographic distribution of the origin of the sampled animals

Figure 1 represents the geographic distribution of the samples (Fig. 1). While the age and gender distribution, in addition to the localization of the lesions sampled are shown in the Tables 1 and 2. The majority of the lesions were localised in the lymph nodes and the lungs (Table 2).

**Table 1:** Age and gender distribution of the sampled animals according to positive and negative cultures

	Male			Female			Total
	<1 year	1–3 years	>3 years	<1 year	1–3 years	>3 years	
Culture +	6	153	20	1	3	66	249
Culture -	4	40	6	2	3	23	78
Total	10	193	26	3	6	89	327

**Table 2:** Localisation of the collected lesions, in addition to the specifications of the animals sampled

Lesion localisation	*n*	Gender		Breed		
		F	M	I	C	L
Liver	5	1	4	0	5	0
Lungs	4	2	2	0	4	0
Lungs and diaphragm	1	0	1	0	0	0
LN	185	45	140	67	117	8
LN and diaphragm	4	1	3	1	3	0
LN and liver	4	1	3	1	3	0
LN and lungs	70	30	40	35	35	2
LN, lungs, and diaphragm	6	1	5	4	2	0
LN, lungs, and liver	18	7	11	4	14	0
LN, lungs, liver, and diaphragm	24	4	20	21	2	1
LN, lungs, liver, and kidney	2	2	0	0	2	0
LN, lungs, pleura, and pericardium	2	2	0	0	2	0
Miliary tuberculosis	2	2	0	0	2	0
Total	327	88	229	133	191	11

*LN* Lymph nodes, *I* Imported, *C* Crossed, *L* Local

The majority of the sampled animals were males between 1 and 3 years and females more than 3 years (Table 1).

### MrpoB PCR

A total of 68% (*n* = 147) of the 215 samples which were AF positive were confirmed as MTBC strains using MrpoB PCR. Thirty two per cent (*n* = 69) of animals were negative for MrpoB.

### Deletion typing

The samples confirmed as MTBC strains were all positive to RD9 deletion typing and then confirmed to be not *M. tuberculosis* strains. A total of 144 (1.7%) of the samples were confirmed to be *M. bovis* as they were positive to the RD4 pcr. Three samples were negative for RD4.

Out of the total of the confirmed *M. bovis* samples, 30% were female while 70% were male. The predominant breed in the positive animals was Crossbreed prime Holstein.

### Spoligotyping

A total of 136 analysed samples were lacking spacers 39 to 43. Forty one different spoligopatterns were found. The most frequent patterns were SB0121, SB0265, SB0120, with frequencies of 24.3% (*n* = 33). 16.9% (*n* = 23), 9.6% (*n* = 13) respectively. They were shown to belong to BOV-1 family. Two other spoligotypes were found in 9 and 6 samples respectively, SB0125 and SB0869. Three spoligotype patterns had no SIT reference on the SITVIT database and were designated as orphan. Ten isolates presented nine undescribed spoligotypes, which were submitted to *M. bovis* website (www.mbovis.org) (Table 3). The discrimination power of spoligotyping, calculated using Hunter and Gaston’s formula was D = 0.9057 [35].

**Table 3:**
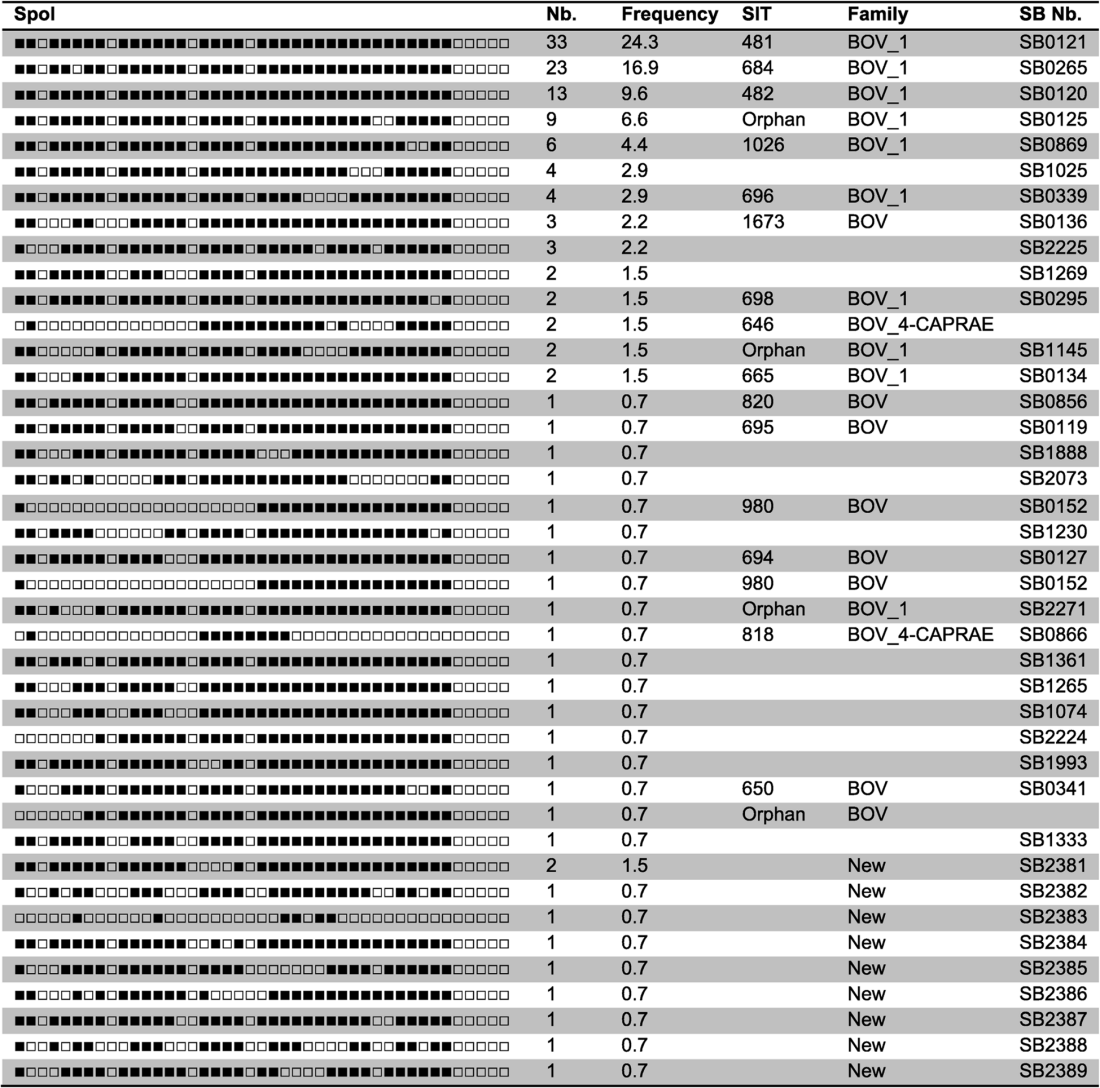
The different spoligotypepatterns of the analysed samples and their relative frequencies

## Discussion

To our knowledge, this is the first study investigating the molecular characterization of bovine tuberculosis among cattle in Morocco. The prevalence of the gross visible lesions suggesting BTB in Rabat and El Jadida slaughterhouses (3.7%) was low compared to the prevalence found previously using tuberculin skin test in the last national survey performed in 2004 [20]. This prevalence was close to those observed in Mali (1.8%) and in Algeria (3.6%).

Surprisingly, no non-tuberculous mycobacteria was found, while in the African context, among slaughterhouse gross visible lesions, 3.3% in Mali [36], and 9% in Burkina Fasso [37] were confirmed as non-tuberculous mycobacteria. This was also observed in Chadian slaughtered cattle [38, 39].

Almost 52.4% of the overall sample size was confirmed to be *M. bovis*, three samples were negative to RD4. While two spoligotypes were typical for the caprae family, the third sample was a new spoligotype. Studies in other Countries (e.g. Nigeria, Ethiopia) found *M. tuberculosis* in cattle [40], whereas in Morocco, remarkably, we didn’t find this MTBC species in our samples.

The most predominant spoligotype pattern found in Morocco is SB0121, belonging to the family BOV 1, this spoligopattern was already reported in Algeria [41], in Tunisia [42] and in Spain [43]. The second most frequent spoligotype was SB0265 which was reported as the second most frequent in the United States from a set of strains collected between 1989 and 2013 [44], in addition, this spoligotype was isolated in Tunisia from a strain coming from Morocco [33], and was also reported in many European countries [45, 46], as well as in Taiwan [47]. The spoligopattern SB0120 had the frequency of 11.7% in our sample size and was reported previously in France from a sample originating from Morocco (SITVIT database), it was also reported in many African countries like Algeria [41], Tunisia [42] and Zambia [48] as well as in Argentina and Spain [43, 49]. Our study shows no similar spoligotype pattern with Mali, indicating that the Sahara desert and the long distance between cattle rearing areas (cattle are only kept until a Latitude of 12 degrees in Mali) seem to be an effective barrier for the transmission of *M. bovis* and/or that there is probably little trade of cattle between Morocco and Mali [36].

Two spoligotypes of three samples belonged to the *M. caprae* family, of which one was found as well in *M.bovis* database as SB0866, this spoligotype was already reported in Spain from one goat, one cattle and one pig in 2011 [50].

The similarities found in some spoligopatterns between Morocco and Algeria could be explained by the shared borders between the two countries, in addition some patterns found in Morocco were previously reported in the United States and in Argentina, two countries from where Morocco have previously imported cattle.

Spoligotypes of African 1 and African 2 clonal complexes were not found among our characterized isolates [51, 52]. African 1 is localized in West and Central Africa, and African 2 is localized in East Africa, consequently, the desert of Morocco could be considered as a potential efficient barrier preventing the introduction of BTB to Morocco from West, Central and East Africa.

The relatively low prevalence of proven *M. bovis* infection in two Moroccan abattoirs has to be interpreted with caution. Firstly, the abattoir prevalence reflects more the prevalence in young bulls and old cows rather than the whole population. Secondly, not all granuloma yielded a bacteriological isolate, hence the true prevalence may be much higher than the one observed. Overall, our findings reflect rather the epidemiological situation of a low level endemic transmission similar to other African countries rather than the one of a peri-urban intensive system [53, 54]. More research is needed to further characterize the ongoing transmission patterns of bovine tuberculosis in view of the development of a locally adapted elimination strategy of bovine tuberculosis in Morocco [55].

## Conclusion

This study presents the first molecular characterization of BTB isolates from Moroccan cattle. *M. bovis* represented a high amount of granulomatous lesions detected in the abattoirs of Rabat and El Jadida. Spoligotype suggests a link of Moroccan BTB to Europe, rather than to Africa, highlighting then the potential of the Moroccan desert barrier for BTB introduction to Morocco from sub-Saharan Africa. Further investigations of BTB strains using new molecular techniques such as whole genome sequencing are needed to clarify more the potential links between Moroccan BTB strains and those of Europe and other African countries.

## Abbreviations

BTB: Bovine tuberculosis
LJ: Lowenstein Jensen
LJG: Lowenstein Jensen with glycerol
LJP: Lowenstein Jensen with pyruvate
MTBC: Mycobacterium tuberculosis complex
NTM: Non-tuberculous mycobacteria
PCR: Polymerase chain reaction
RD: Region of difference
TB: Tuberculosis
ZN: Ziehl-Neelsen
